# Stable isotope analysis in food web research: Systematic review and a vision for the future for the Baltic Sea macro-region

**DOI:** 10.1007/s13280-022-01785-1

**Published:** 2022-10-21

**Authors:** Elvita Eglite, Clarissa Mohm, Jan Dierking

**Affiliations:** 1 Marine Ecology, GEOMAR Helmholtz Centre for Ocean Research Kiel, Düsternbrooker Weg 20, 24105 Kiel, Germany; 2 Department of Forestry and Natural Resources, Purdue University, 715 West State Street, West Lafayette, IN 47907 USA

**Keywords:** Ecosystem-based management, Food web structure and function, Isobank, Knowledge synthesis, Stable isotope ecology, Trophic interaction

## Abstract

Food web research provides essential insights into ecosystem functioning, but practical applications in ecosystem-based management are hampered by a current lack of knowledge synthesis. To address this gap, we provide the first systematic review of ecological studies applying stable isotope analysis, a pivotal method in food web research, in the heavily anthropogenically impacted Baltic Sea macro-region. We identified a thriving research field, with 164 publications advancing a broad range of fundamental and applied research topics, but also found structural shortcomings limiting ecosystem-level understanding. We argue that enhanced collaboration and integration, including the systematic submission of Baltic Sea primary datasets to stable isotope databases, would help to overcome many of the current shortcomings, unify the scattered knowledge base, and promote future food web research and science-based resource management. The effort undertaken here demonstrates the value of macro-regional synthesis, in enhancing access to existing data and supporting strategic planning of research agendas.

**Supplementary Information:**

The online version contains supplementary material available at 10.1007/s13280-022-01785-1.

## Introduction

Food webs mediate major processes as well as pressures in marine ecosystems and provide a functional link from individuals and populations to ecosystem functioning, and ultimately, ecosystem services (Eero et al. 2021). Food web research is therefore essential for our understanding of the performance of individuals, species, and the functioning and trajectories of entire ecosystems and provides a foundation for ecosystem-based management (Thrush and Dayton 2010). It is also a key component of assessments of environmental status, such as good environmental status (GES), under the EU Marine Strategy Framework Directive (MSFD) Descriptor 4 (EU 2008; Korpinen et al. 2022) or Holistic Assessments of the Baltic Sea (HOLAS) by the Baltic Marine Environmental Protection Commission (HELCOM 2018). At the same time, recent analyses have highlighted substantial barriers hampering the systematic application of food web knowledge in practice, one of the main issues being the current lack of synthesis of the large but often scattered knowledge base (Eero et al. 2021). This reflects a global “synthesis gap” and an increased realization of the value of knowledge synthesis as scientific output continues to rapidly increase (Pauli et al. 2017; Wyborn et al. 2018).

Baltic Sea food webs are particularly difficult to characterize due to the spatial changes in community composition along the permanent salinity gradient (Ojaveer et al. 2010), high natural temporal variability, and long-term changes caused by pronounced anthropogenic pressures and rapid climate change (Reusch et al. 2018). Over the last century, ecosystem disturbances have included warming, eutrophication, deoxygenation, overfishing, chemical contamination and the decline and subsequent recovery of top-predators (Reusch et al. 2018), as well as the establishment of non-indigenous species (NIS) (Ojaveer et al. 2017). Due to the fact that many of these problems are directly or indirectly transmitted or modulated via food web processes, recent perspectives have emphasized the importance of food web knowledge for the management of challenges and ecosystem-based management in this system (Blenckner et al. 2015; Eero et al. 2021). In this context, the heterogeneous knowledge base about Baltic food webs, with existing information and data frequently scattered and difficult to access, poses a serious challenge. Synthesizing what we do know has thus been highlighted as an essential step to support Baltic Sea food web research and ecosystem-based management (Backer et al. 2010; Snoeijs-Leijonmalm et al. 2017; Koho et al. 2021).

Within the field of food web research, stable isotope analysis (SIA) has become one of the key methods to assess the dietary ecology, trophic positions, niche properties, and interactions of individual species and functional groups as well as energy flows and the structure of food webs (Boecklen et al. 2011; Nielsen et al. 2018). The method is based on the analysis of the stable isotope (SI) composition (the ratio of heavy to light SIs of different elements, commonly denoted as δ-values), of whole bodies or specific tissues of producers and consumers, and ultimately follows the principle “*you are what you eat*” (DeNiro and Epstein 1978, 1981). This means that the SI composition of a consumer’s tissue will reflect the dietary resource use over time, albeit with additional complexity, introduced in particular by animal physiological processes that lead to isotopic fractionation and varying tissue turnover times, and thus, consumer-resource discrimination (Boecklen et al. 2011; Shipley and Matich 2020). The resulting time-integrated view contrasts with the snapshot view of the diet provided by stomach content analysis based on traditional visual identification (Hyslop 1980) or molecular approaches (Pompanon et al. 2012; Nielsen et al. 2018), thus offering unique insights into biological systems. At the same time, as with any method, SIA also entails its own limitations and methodological challenges, including uncertainty in fractionation and overlapping SI values of putative dietary sources (Boecklen et al. 2011; Petta et al. 2020; Matich et al. 2021).

From a method perspective, two SI approaches have become established: bulk SIA (BSIA), in which the isotopic composition of the entire (“bulk”) sample is obtained (DeNiro and Epstein 1981), and compound-specific SIA (CSIA) of individual amino acids (Chikaraishi et al. 2007) or fatty acids (Bec et al. 2011). BSIA applications in ecological studies have become commonplace since the onset of biological applications in the late 1970s, focusing on SI ratios of nitrogen (δ^15^N) (used frequently as measure of trophic position), carbon (δ^13^C), and sulfur (δ^34^S) (e.g., as measure of basal resource and habitat use) (DeNiro and Epstein 1978, 1981; Peterson and Fry 1987), and more recently, oxygen (δ^18^O) and hydrogen (δ^2^H/δD) (used e.g., to track animal natal origins and migrations) (see reviews by Vander Zanden et al. 2016; Shipley and Matich 2020). In contrast, CSIA has gained momentum in food web studies only over the last 20 years focusing almost exclusively on SIs of nitrogen and carbon (McClelland and Montoya 2002; Chikaraishi et al. 2009; Larsen et al. 2009; McMahon and McCarthy 2016), with some first applications of hydrogen SI (Fogel et al. 2016; Pilecky et al. 2021). CSIA has a number of potential advantages over BSIA, including the possibility to determine consumer trophic levels without the need for external baseline samples, and an increased power to differentiate among basal resources or dietary sources. At the same time, CSIA is also more costly and methodologically demanding than BSIA (see review by Nielsen et al. 2018).

The complementarity of SIA with traditional diet analyses and its broad applicability have contributed to the emergence of “SI ecology” as a research field in its own right (Fry 2006) since the method was first applied in ecological studies in the 1970s (Haines 1976; DeNiro and Epstein 1978). Beyond the rapidly growing number of applications in trophic ecology and fundamental food web research, SIA studies now increasingly explore the link between anthropogenic activities and the environmental state. For example, SIA has proven powerful in the identification of the fates of anthropogenic nitrogen (Cabana and Rasmussen 1996), nitrogen fixed by cyanobacteria (Montoya et al. 2002), and eutrophication in marine systems (Voss et al. 2000), impacts of NIS on food web structure (McCue et al. 2020), or the biomagnification of contaminants along food chains (Broman et al. 1992), leading to scientific advances that would have been difficult or impossible to obtain with other methods. As such, SIA has become a method providing food web understanding and supporting the science-based management of marine environmental challenges (Glibert et al. 2019), with a strong potential to support ecosystem assessments (Mack et al. 2020).

The need for knowledge synthesis in the growing field of SI ecology is evident from a mounting number of reviews focusing on specific organism groups such as marine mammals (Newsome et al. 2010) or elasmobranchs (Shiffman et al. 2012), or research topics such as animal migrations (Hobson et al. 2010) or invasion ecology (McCue et al. 2020). Here, we take a different approach and aim to alleviate the synthesis gap in food web research by providing the first macro-regional review of SI applications in ecological studies, focusing on the Baltic Sea region. This includes the compilation of an open-access meta-data collection allowing both experienced SI researchers and newcomers to the field to quickly identify and grasp previous Baltic Sea SIA work on any fundamental or applied research topic, time period, sub-region, taxon, and trophic group of interest. To do so, we reviewed how, when, where, on which taxa, and for which purposes SIA has been applied in the Baltic Sea region, and compared the emerging patterns with the development of the field globally. We then discuss scientific advances resulting from these applications, but also structural shortcomings of the research field in the context of the spatio-temporal characteristics and resource management challenges of the Baltic Sea. We close with a vision on how to overcome these shortcomings via improved collaboration, coordinated sampling efforts, and the systematic use of open-access SI databases, to promote future food web research and science-based resource management and conservation in the Baltic Sea macro-region.

## Materials and methods

### Literature search

We conducted a comprehensive search of peer-reviewed ecological studies that employ SIA in the Baltic Sea region, using the search engines Web of Science, Google Scholar, and ScienceDirect by Elsevier. The search included the terms “Baltic Sea" and “stable isotope", and any of the following: “food web”, “trophic structure”, “trophic cascade”, “trophic interaction”, “food chain”, “compound-specific”, “animal migration”, “zooarchaeology”, “eutrophication”, “organic pollutant”, “heavy metal”, “cyanobacteria”, “phytoplankton”, “macroalgae”, “zooplankton”, “fish”, “seal”, “seabird”, “jellyfish”, “seston”, “seagrass”, “non-indigenous species (NIS)”, “invasive species”, “benthic”, “pelagic”. We also applied the same search string for the individual Baltic Sea sub-basins (e.g., "Bornholm Basin”, “Gotland Basin”, “Bothnian Bay,” “Bothnian Sea,” “Kiel Bay,” “Gulf of Gdansk,” etc.) instead of the common term “Baltic Sea”, and for individual genus names of key Baltic Sea taxa that are the frequent focus of scientific studies (e.g., “*Aphanizomenon*”, “*Nodularia*”, “*Gadus*”, *“Perca”*, “*Temora*”, “*Acartia*”, “*Limecola,*” “*Zostera*”, “*Fucus*”) instead of trophic group names. All identified articles were cross-checked for additional relevant references. We also searched the online open data repositories *Dryad* and *Pangaea* for data sets with corresponding peer-reviewed studies fitting the scope of this review. The final search was conducted on July 10, 2021.

### Screening of studies for fit with the review

All identified studies were screened, and only those fitting the scope of the review were included in subsequent formal analyses. The inclusion criteria included (1) geographic focus on the Baltic Sea region, (2) focus on marine or brackish, but not freshwater (lake or stream) systems, (3) application of SIA, and (4) ecological focus. Under (3), studies applying BSIA and CSIA were included, whereas studies applying radiocarbon (^14^C) labeling (e.g., Engström et al. 2000, van de Bund et al. 2001) or other radioactive isotopes (e.g., Zalewska and Suplińska 2013) were excluded. Moreover, under (4), studies focusing on biogeochemistry and microbial processes including denitrification, nitrification, anaerobic ammonium-oxidation (e.g., Hietanen et al. 2012; Dalsgaard et al. 2013) or nitrogen fixation rates (e.g., Wasmund et al. 2001), marine geology, geophysics, and geochemistry (e.g., Scheurle and Hebbeln 2003) were excluded. Gray literature was excluded from all formal analyses but is listed in Supplementary Table S4.

### Extraction of study parameters

**Fig. 1:**
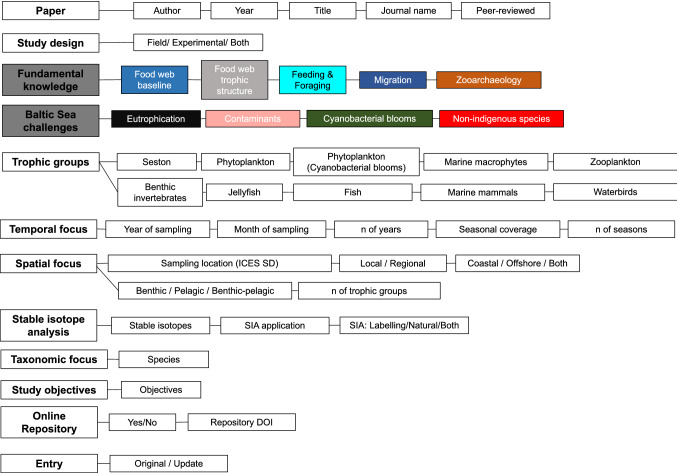
Schematic view of the structure and content of the meta-data table containing the extracted information from all 164 studies in this review. The table is structured into 12 overarching categories (boxes on the left) and 43 individual parameters (boxes on the right with connecting lines to overarching category). Dark gray boxes highlight the two main categories of fundamental and applied research topics; colors of the boxes under these categories correspond to the topic colors in Figs. 2 and S1. *Note*: The corresponding meta-data collection for all 164 studies is available open-access in the Dryad repository under the link: https://datadryad.org/stash/dataset/doi:10.5061/dryad.sj3tx966d

We extracted the parameters defining the focus of each study along multiple dimensions (e.g., spatial, temporal and taxonomic focus, topic, study design). All extracted meta-data were compiled in a table (“meta-data collection” in the following), structured into 12 overarching categories and 42 parameters, each as a separate column header, grouped within these categories (Fig. 1, Table S1). The title, keywords, abstract, and full text of all studies were intensively screened to ensure consistent extraction and classification, and clear classification rules were established for all parameters (Table S1).

### Categorization of studies by scientific topic

We categorized studies by their scientific topics in a two-step procedure. First, all studies were assigned to at least one fundamental research topic under the category “Fundamental knowledge”, with the topic areas: “Food web baseline”, “Food web trophic structure”, “Feeding and Foraging”, “Migration”, and “Zooarchaeology”. All of these topics were considered to have a primary focus on food web research, except “Migration” and “Zooarchaeology”, which contain information relevant for food web research but have a different primary focus. In a second step, where applicable, studies were assigned to applied topics (i.e., explicitly related to anthropogenic pressures in the Baltic Sea). Under the header “Baltic Sea challenges”, this included the topic areas: “Eutrophication”, “Contaminants”, “Cyanobacterial blooms”, and “NIS”. Accordingly, all studies were grouped into at least one topic under “Fundamental knowledge”, and where applicable, into one or more additional topics addressing “Baltic Sea challenges”.

The categorization of studies was based primarily on the article keywords and abstract, and checked for consistency by in-depth scrutiny of each article. For example, regarding the category of “Fundamental knowledge”, studies tracing allochthonous nutrient inputs at the base of the food web were grouped under the topic "Food web baseline", studies that investigate multiple trophic interactions under the topic “Food web trophic structure”, and studies that investigate the feeding ecology of individual species under the topic "Feeding and Foraging".

### Availability and use of meta-data collection

The resulting meta-data collection contains all extracted parameters of the 164 peer-reviewed SIA studies with an ecological focus in the Baltic Sea region identified in this systematic review, but does not contain primary SI datasets. We used this collection, applying filtering and Pivot table functions in Excel, to extract summary statistics and to cluster studies for this review. The meta-data collection is available open-access in the online repository *Dryad* (Eglite et al. 2022) under the link https://datadryad.org/stash/dataset/doi:10.5061/dryad.sj3tx966d, and can be used to quickly grasp and access all previous Baltic Sea SIA work as foundation for future SIA studies.

## Results

### Timeline of published stable isotope studies

**Fig. 2:**
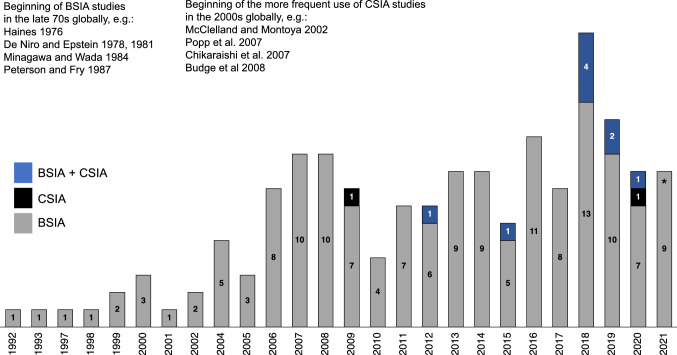
Timeline of the publication of stable isotope ecology studies in the Baltic Sea (*n* = 164). For reference, examples of highly cited foundation studies that initiated or substantially advanced the bulk stable isotope analysis (BSIA) and compound-specific stable isotope analysis (CSIA) research fields globally are provided. *Only studies published until July 10, 2021 included

Our systematic review identified 164 peer-reviewed SIA studies addressing ecological topics in the Baltic Sea, with a strong increase in the number of studies published per year over time (Fig. 2, Table S2). Of these studies, 153 applied BSIA, nine both BSIA and CSIA, and two exclusively CSIA. The first BSIA study was published in 1992 (Broman et al. 1992) and applications became more routine by the 2000s, whereas the first CSIA study was published in 2009 (Glaubitz et al. 2009) and applications became more common only over the last decade. For both BSIA and CSIA, the comparison with key studies in the field globally showed a time lag of ca. 10–15 years in the development of SIA studies in the Baltic Sea ecology field (Fig. 2). Out of 126 studies published since 2008, the first year in which the widely used online open-access repositories *Dryad* (released 2008) and *Pangaea* (released 1995) were both operational, only 10 had submitted the corresponding primary data sets to these repositories (eight in *Dryad*, two in *Pangaea*), the first one being Mittermayr et al. (2014).

### Spatial coverage of SIA studies

**Fig. 3:**
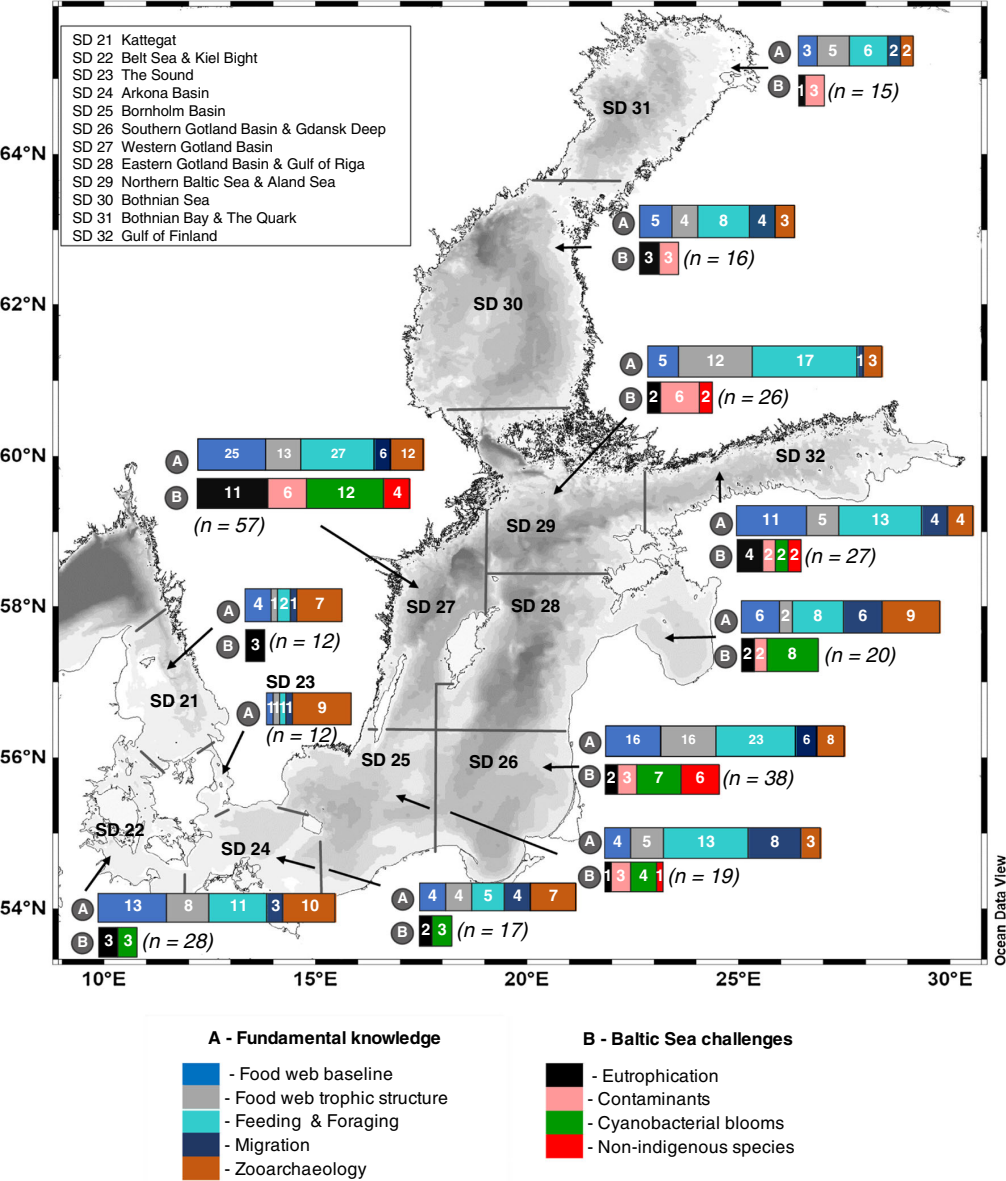
Spatial coverage and scientific topics of stable isotope ecology studies in the Baltic Sea (*n* = 164), by primary focus on "Fundamental knowledge" (bars denoted “A”) and "Baltic Sea challenges" (bars denoted “B”) and International Council for the Exploration of the Sea (ICES) sub-divisions (SDs). Notes: Studies may fall under more than one topic and thus the cumulative number of studies under all topics is higher than the total number of studies. “Migration” studies are assigned to SDs based on sampling location, although migration routes can extend to other SDs. The total number of studies per SD is displayed on the map in italics (*n* = study number). For the complete list of publications underlying the map and classification, see Table S2 and the open-access meta-data collection https://datadryad.org/stash/dataset/doi:10.5061/dryad.sj3tx966d

In combination, published SIA studies have covered all sub-regions of the Baltic Sea (Fig. 3). At the same time, there were large sub-regional differences, with stronger coverage of ICES sub-division (SD) 22 (Belt Sea, Kiel Bight), SDs 26, 27, 29 (central Baltic Sea), and SD 32 (Gulf of Finland), but weaker coverage of SDs 21, 23, and 24 in the western, SD25 in the central, SD 28 in the eastern, and SDs 30 and 31 in the northern Baltic Sea. Some spatially confined areas were the focus of a large number of studies, including three notable “hotspots”, Himmerfjärden Bay (*n* = 17; SD 27), Curonian Lagoon (*n* = 9; SD 26), and Puck Lagoon/Puck Bay (*n* = 6; SD 26).

### Research topics covered by SIA studies

SIA studies in the Baltic Sea addressed a broad range of fundamental research questions in ecology, most of them on food web-related topics (Fig. 3, panel A “Fundamental knowledge”). Moreover, close to half of these studies (*n* = 77) also addressed applied questions related to anthropogenic pressures (Fig. 3, panel B “Baltic Sea challenges”; Table S2).

Under “Fundamental knowledge,” the topic covered most was “Feeding and foraging” (*n* = 89), followed by “Food web baseline” (*n* = 66) and “Food web trophic structure” (*n* = 50), whereas “Zooarchaeology” (*n* = 24) and “Migration” (*n* = 17) were covered less (Fig. S1A). Under “Baltic Sea challenges”, the topic covered most was “Eutrophication” (*n* = 27), followed by “Cyanobacteria blooms” (*n* = 25) and “Contaminants” (*n* = 22), whereas “NIS” were covered by only 12 studies (Fig. S1B).

The relative importance of topics differed among areas. For example, whereas the fundamental knowledge topic “Feeding and foraging” was important in all areas of the Baltic Sea, other topics were represented over-proportionally in specific areas, e.g., “Food web trophic structure” in ICES SD 26, “Zooarchaeology” in SD 27 or “Migration” in SD 25 (Fig. 3). Under Baltic Sea challenges, the topic “Eutrophication” played an important role in most SDs, whereas the topic “Contaminants” was covered primarily in the central and northern but not the western Baltic Sea, and “Cyanobacterial blooms” in the western and central but little in the northern areas Bothnian Sea (SD 30) and Bothnian Bay (SD 31), where cyanobacteria blooms are also less common. Regarding NIS, studies were mainly concentrated on SD 26 and SD 27, with little or no studies in the rest of the Baltic Sea.

The relative importance of applied topics under “Baltic Sea challenges” also shifted over time. While “Contaminants” were the most addressed topic in the period up to 2010 (43% of studies), this number declined to 17% for the period since 2010. In contrast, “Eutrophication” remained consistently important (29% of studies prior to 2010 versus 33% afterward) and the topics “NIS” (7% versus 17%) and “Cyanobacteria blooms” (21% versus 33%) increased in importance (Fig. S3B).

In contrast, the relative importance of fundamental knowledge topics remained remarkably constant, with the exception of a decline in the importance of the topic “*Zooarchaeolog*y” from 16 to 7% and slight increases in the topics “*Migrations*” from 5 to 8% and “*Food web baselines*” from 22 to 29% over time (Fig. S3A).

### Main trophic groups and key species studied

**Fig. 4:**
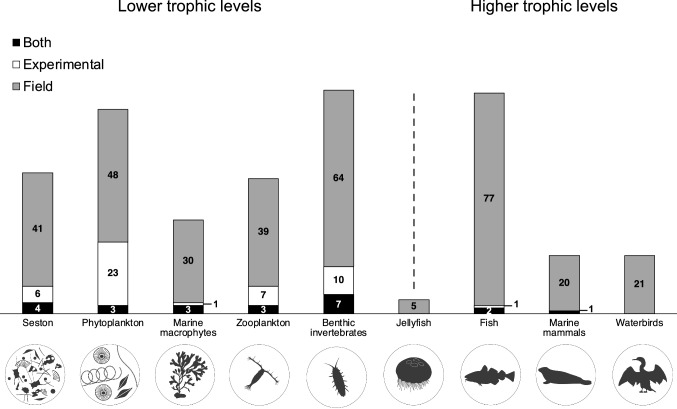
The coverage of trophic groups in stable isotope ecology studies in the Baltic Sea. Numbers in the bars represent the count of studies from the overall study pool (*n* = 164) providing data for specific groups. Stacks represent field, experimental, and combined field and experimental studies (“both”). “Jellyfish” can be placed between lower and higher trophic levels, in being part of the plankton and also consuming plankton at different trophic levels. For more detailed inclusion criteria for the different trophic groups, see Table S1

SIA studies have covered all of the major trophic groups from both lower and higher trophic levels of the Baltic Sea food webs (Fig. 4), with most individual field studies focusing on either one or two trophic groups (67%), compared to 33% including data for organisms from three or more trophic groups (Table 1). On higher trophic levels, most studies focused on fish (*n* = 80), whereas marine mammals (*n* = 21) and waterbirds (*n* = 21) were covered less. On intermediate and lower trophic levels, benthic invertebrates (*n* = 81), zooplankton (*n* = 49), and phytoplankton (*n* = 74) were studied most frequently, whereas jellyfish were covered by only five studies to date.

**Table 1:** Study design of stable isotope ecology studies in the Baltic Sea (including both field and experimental studies, upper part of the table) and the specific study foci of field studies (lower part of the table). Numbers reflect counts of studies. Notes: The combined counts of studies under specific parameters can differ from the total number of field studies, because archaeological studies do not fit categorizations for several parameters (marked by *) and because some contemporary studies omitted technical details needed for the categorization. The combined count under “Seasons” is higher than the total number of field studies because studies including more than one season are counted repeatedly. “Both” under “Study design” refers to studies that include both field and experimental work

All studies		*n*
Study design	Total	164
	Field	134
	Experimental	19 (labeled SI—11)
	Both	11 (labeled SI—2)

Field studies		*n*
Spatial focus	Local	90
	Regional	43
Temporal focus*	1-year	45
	≥ 2-years	58
	1 season	51
	2 seasons	34
	3 seasons	7
	4 seasons	6
Season*	Summer	76
	Spring	38
	Autumn	29
	Winter	21
Habitat*	Coastal	72
	Both	23
	Offshore	14
	Benthic-Pelagic	40
	Benthic	39
	Pelagic	31
Trophic groups	1 group	56
	2 Groups	34
	3 Groups	22
	≥ 4 Groups	22

**Fig. 5:**
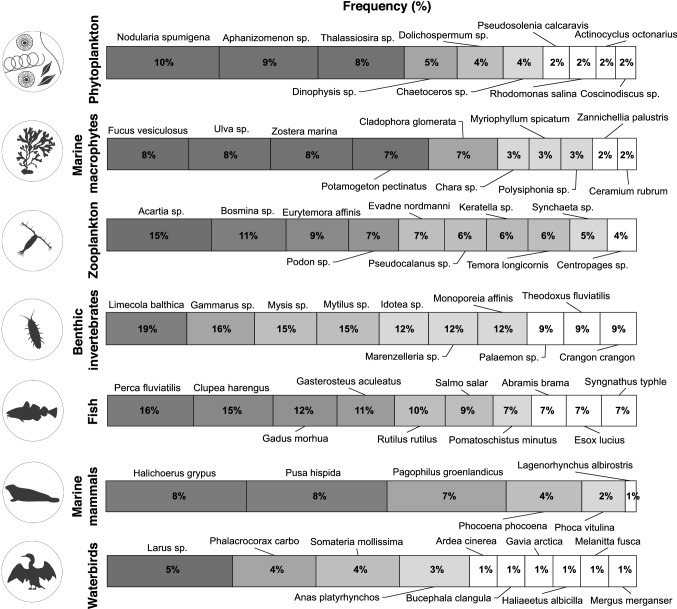
The coverage of individual species within seven major trophic groups in stable isotope ecology studies in the Baltic Sea, based on the proportion of studies in the overall study pool (*n* = 164) addressing the respective species. The 10 species with the highest coverage within each respective trophic group are included; all species are thus included if a group comprised less than 10 species. “Jellyfish” – excluded due to a low number of studies. For lists of individual taxa covered and the corresponding total number of unique taxa, as well as the count of studies covering each taxon, see Table S3

Within these broad trophic groups, SIA values for a total of more than 240 individual species were provided. This includes 75 benthic invertebrate, 52 fish, 44 phytoplankton, 24 zooplankton, 19 waterbird, 16 marine macrophyte, six marine mammal, and three jellyfish species (Fig. 5, Table S3). Only a relatively small proportion of these species was covered regularly, whereas isotopic values were reported only once or for infrequent time-points and dispersed locations for the majority of species. Specifically, only the seven species *Acartia* spp.*, Limecola balthica, Gammarus* sp.*, Mysis* sp., *Mytilus* sp.*, Perca fluviatilis,* and *Clupea harengus* were covered by more than 20 SIA studies in the Baltic Sea region, whereas 164 species were covered by five or less studies to date (Fig. 5, Table S3).

### Study design of published SIA studies in the Baltic Sea

The large majority of the 164 SIA studies in the Baltic region were exclusively field-based (*n* = 134) and used “natural” SI values (82%), compared to 7% of studies that combined field and experimental work (natural SI: *n* = 9; labeled SI: *n* = 2) and 12% of studies that were exclusively experimental (natural SI: *n* = 8; labeled SI, usually combined with small natural SI component: *n* = 11) (Table 2). From a spatial perspective, only 32% of field studies covered sites from at least two ICES SDs (termed “regional” here), compared to 67% focusing on a single SD (termed “local”), which often included only one or a limited number of sampling locations. In terms of habitats and food web coverage, 66% of field studies focused exclusively on coastal areas, compared to 13% on offshore areas and 21% on both, and 35% of field studies focused exclusively on benthic systems, 28% on pelagic systems, and 36% on both (Table 1). While BSIA studies covered benthic and pelagic systems in roughly equal numbers, CSIA studies focused mainly on pelagic systems (8 of 11 studies) (Table 2).

**Table 2:** Stable isotope technique (bulk stable isotope analysis, BSIA; compound specific stable isotope analysis, CSIA) and specific stable isotopes applied in studies with a benthic, pelagic, or combined benthic-pelagic focus in the Baltic Sea stable isotope ecology field. Note: archeological studies (*n* = 24) were excluded from the counts because the sample sets (e.g., from archaeological middens) often do not allow the categorization as benthic or pelagic study

	Benthic	Pelagic	Benthic-Pelagic	Total
**Bulk**				138
δ^13^C	43	26	44	113
δ^15^N	49	37	46	132
δ^34^S	3	1	6	10
δ^18^O		2	1	3
δ^2^H			1	1
**CSIA**				10
*Amino acids*				
δ^13^C		2	1	3
δ^15^N	2	4		6
*Fatty acids*				
δ^13^C		2		2

From a temporal perspective, 85% of studies had a contemporary focus, whereas 15% used samples from archaeological collections. Of the former, 44% of field studies covered a single year and 56% two or more years (Table 1). Within years, 52% of field studies focused on a single season compared to 42% on two or three and only 6% on four seasons. Summer was covered most (57% of studies) and winter least (16% of field studies).

Finally, from a technical perspective, natural abundance BSIA studies mainly included δ^15^N (96% of BSIA studies) and δ^13^C (82%), and much less so δ^34^S (5%), δ^18^O (2%), and δ^2^H (< 1%) (Table 2, Fig. S2). Regarding CSIA, nine of the 10 studies focused on individual amino acids (six using δ^15^N, three using δ^13^C) and two studies on fatty acids (both using δ^13^C) (Table 2, Fig. S2). Overall, the most common approach was the “traditional” combination of δ^13^C and δ^15^N (60% of studies, Fig. S2), whereas few studies applied more complex combinations of δ^13^C and δ^15^N with δ^34^S (7%) or δ^18^O (one study), and not a single study combined δ^2^H and δ^18^O isotopes (Fig. S2). Moreover, labeled SIs were used in 13 experimental studies (Table 1), of which 10 applied labeled bulk ^15^N, nine labeled bulk ^13^C, two labeled ^15^N amino acid, and one labeled ^13^C fatty acid analysis (Fig. S2).

## Discussion

Food web knowledge is an essential component of ecosystem-based management and assessments of environmental status. However, its practical application is often hampered by the lack of synthesis of the existing knowledge. In the Baltic Sea, SIA has become a pivotal method in the field of food web research, with 164 studies published since the year 1992. Based on the first systematic review of this vast knowledge source, we discuss the substantial advances in fundamental ecological and applied research topics achieved with SIA, but also the structural shortcomings limiting the potential of this research field. We then provide a perspective on how to overcome these current shortcomings via improved collaboration, coordinated sampling efforts, and the systematic use of open-access SI databases, to promote food web research and science-based resource management and conservation in the Baltic Sea macro-region.

### Knowledge gains regarding fundamental ecological topics

The large and rapidly growing number of studies demonstrates that SIA has become an important tool in ecological research on the Baltic Sea system. The range of addressed fundamental ecological topics mirrors the wide applicability of the method that is visible in global approaches (Boecklen et al. 2011; Twining et al. 2020). Here, we highlight key SIA applications and knowledge gains for each topic.

*Feeding and foraging *Information on the feeding ecology and diet of animals is essential to understand their performance (i.e., condition, reproductive output, and ultimately population trends) and role in food webs. SIA has become one of the principal methods providing this information (Nielsen et al. 2018). This is reflected by the large proportion of Baltic SIA studies addressing this topic, and by the wide range of addressed species, including otherwise little assessed non-commercial (e.g., various fish species, crustaceans) and NIS (see Section *Non-indigenous species*), commercial fish species including herring (Gorokhova et al. 2005) and salmon (Torniainen et al. 2017a), as well as marine mammals (e.g., Angerbjörn et al. 2006) and waterbirds (e.g., Morkune et al. 2016). Examples of applications include comparisons of the spatio-temporal dietary overlap and extent of competition, e.g., between the non-indigenous round goby and native fish species including flounder (Karlson et al. 2007), cod and perch (Almqvist et al. 2010), and benthic fishes (Herlevi et al. 2018). Dietary studies combining SIA and stomach content analysis allowed insights regarding both the trophic niche and the specific diet composition of species (e.g., Lehtiniemi et al. 2009). Moreover, feeding strategies under physiological stress were assessed in crustaceans (particularly mysids) in both experimental (e.g., Gorokhova and Hansson 1999) and field studies (Ogonowski et al. 2013).

*Food web baseline* Identifying basal organic matter sources is essential for the understanding of food webs (Layman et al. 2012), and particularly relevant in the light of structural food web changes under anthropogenic and global change (Kortsch et al. 2015; Fey et al. 2021). Here, SIA can be particularly powerful, because organic matter sources often differ substantially in their SI values, with δ^13^C as a powerful tracer of terrestrial versus marine production, δ^15^N of different nitrogen sources, and δ^34^S of benthic versus pelagic production (Peterson 1999; Layman et al. 2012). In the Baltic Sea, specific foci have included the role of allochthonous terrestrial (e.g., linked to river input and run-off) versus autochthonous marine production (e.g., Rolff and Elmgren 2000; Bartels et al. 2018), benthic–pelagic coupling (e.g., Kiljunen et al. 2020), the distribution and fate of δ^15^ N-enriched discharges from sewage treatment plants particularly in coastal ecosystems (e.g., Savage 2005; Schubert et al. 2013), as well as the role of N_2_ fixation by cyanobacteria (see Section *Cyanobacteria blooms*), and changes related to anthropogenic pressures (Bianchi et al. 2000).

*Food web trophic structure* One of the globally applied advantages of SIA is the ability to reveal the trophic position of organisms and the trophic structure of food webs due to the enrichment of heavier isotopes, in particular ^15^N, from one trophic level to the next (Layman et al. 2012). In the Baltic Sea, changes in food web structure have been of particular interest due to strong vectors of change and the occurrence of regime shifts (Reusch et al. 2018). Our review showed an impressive coverage of all major trophic groups and individual species within these groups by SIA studies. Knowledge gains included insights regarding the trophic positions and functional roles of various food web “players”, including the commercially and ecologically important consumer cod (Deutsch and Berth 2006), species of conservation relevance such as marine mammals and birds (Hobson et al. 2015), NIS (see Section *Non-indigenous species*), and foundation species such as eelgrass (Jankowska et al. 2018). Other foci were the pelagic food web structuring along the Baltic Sea salinity gradient (e.g., Larsen et al. 2020) and the magnitude of benthic–pelagic coupling in this shallow sea (Bartels et al. 2018; Kiljunen et al. 2020).

*Migration *Different habitats (e.g., benthic versus pelagic, coastal versus offshore) as well as geographic areas oftentimes diverge in isotopic baselines. This has made SIA a tool of choice to identify the migration patterns of animals, because the time-integrated SI signature acquired by feeding in one area remains visible in an animal’s tissue after the migration to a new area (Hobson 1999). Baltic Sea SIA studies have generated substantial knowledge about the horizontal migration patterns of Atlantic salmon (Torniainen et al. 2014; Orell et al. 2018), but surprisingly, have not addressed other migratory fishes such as seatrout or coregonids. Other foci have been vertical migration strategies of crustaceans, particularly diurnal migrations of mysid populations (Ogonowski et al. 2013). The concept of using maps of isotopic variation (termed “isoscapes”, short for “isotopic landscape”), to track animal migration (Hobson et al. 2010) is gaining attention in the Baltic Sea (Torniainen et al. 2017b), but has not yet been addressed as systematically as in many other geographic areas.

*Zooarchaeology *SIA analysis of archived samples, such as collagen in fish otoliths (Grønkjær et al. 2013), animal bones (Schoeninger and Moore 1992), bird feathers (Gagne et al. 2018) or fossils from sediment cores (van Hardenbroek et al. 2018), provides insights into the biological past, including animal diets and migrations and long-term ecosystem changes (Pilaar Birch 2013). In the Baltic Sea, “SI zooarchaeology” remains underexplored, but studies focusing primarily on past human diets frequently include SI data of Baltic Sea fauna, mainly of fish and marine mammals. Moreover, recently archived eel bone samples from freshwater, brackish, and marine habitats were used to establish baseline values for human diet studies (Robson et al. 2012). Archeological research also addressed fish trading and commercialization in the Baltic and North Sea regions (Barrett et al. 2008, 2011; Orton et al. 2011), and provided δ^13^C and δ^15^N values of cod bone samples from geographically diverse medieval settlements, thereby providing information on spatial isotopic variability. Finally, BSIA of cyanobacterial pigments (Bianchi et al. 2000; Borgendahl and Westman 2006; Szymczak‐Żyła et al. 2019) and cladoceran fossils (Struck et al. 1998) in sediment cores were used to investigate the role of cyanobacteria-produced nitrogen over the last century and during the Baltic Sea formation in the Holocene. The historic perspective (decades to millenia) provided by these studies represents a unique counterpart to the more recent (years to decades) perspective of contemporary SIA studies. In contrast to most contemporary SI data, paleo-dietary data are partly available open-access via the dIANA database (Etu-Sihvola et al. 2019).

### Knowledge gains regarding Baltic Sea challenges

Eutrophication, cyanobacteria blooms, contaminants, and NIS are among the most pervasive resource management challenges in the heavily anthropogenically impacted and rapidly changing Baltic Sea (Reusch et al. 2018). The substantial insights regarding these four challenges provided by 70 SIA studies to date are addressed individually below.

*Eutrophication *Eutrophication is a global environmental concern (Rabalais et al. 2009) and considered the single most harmful anthropogenic pressure in the Baltic Sea (HELCOM 2018; Bonsdorff 2021). The efficient management of eutrophication requires an understanding of the sources and fates of nutrients, for which δ^15^N is a powerful tracer (Cabana and Rasmussen 1996; Hastings et al. 2013). This tracer role has been advanced and exploited in the Baltic Sea by multiple studies, both for nitrogen from sewage and manure (e.g., Hansson et al. 1997; Savage 2005; Schubert et al. 2013) and fixed by cyanobacteria (see Section *Cyanobacteria blooms*), and has been instrumental for the understanding of structural and functional changes of Baltic Sea food webs, e.g., via bottom-up effects (see Section *Food web baselines*). Recent SIA studies continue to emphasize the far-reaching and long-lasting consequences of eutrophication (Golubkov et al. 2019; Liénart et al. 2021).

*Cyanobacterial blooms *Cyanobacteria blooms are projected to increase in warming seas (Visser et al. 2016), enhancing atmospheric N_2_ fixation and undermining efforts to curb eutrophication (Voss et al. 2011). In the rapidly warming Baltic Sea, SI studies have revealed that nitrogen fixed by cyanobacteria is efficiently transferred through food webs and plays a substantial role for secondary production (see review by Karlson et al. 2015a). In this context, the depleted bulk δ^15^N values (Rolff 2000) and characteristic CSIA signatures (Loick-Wilde et al. 2012) of nitrogen fixed by cyanobacteria, and of labeled SI in experimental approaches (Adam et al. 2016) have been advanced and used to great effect by the Baltic SI ecology community. In particular, BSIA helped to elucidate the role of microbial food webs and zooplankton grazing strategies (e.g., Rolff 2000; Eglite et al. 2018; Motwani et al. 2018) as well as benthic feeders (e.g., Limén and Ólafsson 2002; Karlson et al. 2014) in the transfer of diazotrophic nitrogen, whereas CSIA was used to elucidate the role of complex biochemical processes and cyanobacterial supply of de novo synthesized amino acids for planktonic food webs (e.g., Loick-Wilde et al. 2012, 2018b; Eglite et al. 2019). Moreover, SIA studies addressed cyanobacterial toxins in fish (e.g., Lesutienė et al. 2018) and neurotoxins in coupled pelagic–benthic food webs (Zguna et al. 2019).

*Contaminants *Contamination with persistent organic pollutants and heavy metals has been another core environmental problem in the Baltic Sea region (HELCOM 2018; Reusch et al. 2018), including potential health risks from the consumption of commercial fish species. Contaminant concentrations in biota can vary depending on the contaminant levels in the environment and the extent of bioaccumulation along food chains (Tuomisto et al. 2020). The application of SIA can promote functional understanding by providing trophic level information and quantitative estimates of bioaccumulation for contaminant studies, a concept that was pioneered in the Baltic Sea (Broman et al. 1992; Rolff et al. 1993). Since then, a growing number of studies have advanced our knowledge of organic pollutant accumulation, with a particular focus on commercial fishes like Atlantic salmon (e.g., Berglund et al. 2001; Nfon et al. 2009; Vuori et al. 2012) or perch (e.g., Hanson et al. 2020; Suhareva et al. 2021), but also other top consumers like Eider ducks (Broman et al. 1992). Fewer studies have addressed entire fish communities (Burreau et al. 2004, 2006) or pelagic and benthic food webs (Nfon et al. 2008), including two studies addressing mercury biomagnification (Nfon et al. 2009; Jędruch et al. 2019). The decreasing proportion of SIA studies on contaminants over time in the Baltic may reflect a more balanced focus across challenges, including also NIS and cyanobacteria blooms.

*Non-indigenous species* NIS are introduced and become established at unprecedented rates, constituting a major environmental problem globally (Early et al. 2016) and in the Baltic Sea (Ojaveer et al. 2017). A major question regarding NIS concerns their impact on native species and food webs, which is often linked to feeding ecology (Ojaveer et al. 2021). As discussed in the Section *Feeding and foraging*, the most common application of SIA in the Baltic Sea has been the elucidation of the dietary ecology of various species, including 11 NIS studies. These studies provided new insights regarding NIS trophic positions and niches and effects on whole food web structuring, e.g., of round goby *Neogobius melanostomus* (e.g., Karlson et al. 2007; Herlevi et al. 2018; Rakauskas et al. 2020), the predatory cladoceran *Cercopagis pengoi* (Gorokhova et al. 2005; Holliland et al. 2012), the polychaete *Marenzelleria arctia* (Karlson et al. 2015b), as well as amphipods and mysids (Berezina et al. 2017). These applications have demonstrated the particular usefulness of SIA, as a time-resolved method requiring lower sample sizes than traditional stomach content analysis (Nielsen et al. 2018), for NIS studies with their often low available sample sizes.

### Structural shortcomings limiting the potential of Baltic Sea SIA work

Despite the wealth of new information provided by SIA studies, patterns revealed by the systematic review also suggest that the full potential of this method in Baltic Sea ecological and food web research has not been reached. In the following, we identify current structural shortcomings and provide a perspective on how to address them.

*Shortcoming 1: Limited spatio-temporal and taxonomic scope and resolution* Considered jointly, the 164 SIA studies in the Baltic Sea offer an impressive spatial, temporal, trophic group, and species coverage of the Baltic Sea. Conversely, considered individually, few studies achieve complete seasonal coverage (but see Rolff 2000; Jaschinski et al. 2011), interannual comparisons (but see Nordström et al. 2009), joint coverage of low and high trophic levels (but see Thormar et al. 2016; Corman et al. 2018), or spatial coverage spanning multiple replicate sites from different sub-regions (but see Loick-Wilde et al. 2018a; Orell et al. 2018). Even fewer studies combine several of these dimensions (but see Nadjafzadeh et al. 2016; Marcelina et al. 2018; Kiljunen et al. 2020), and no single study achieves simultaneous high-resolution coverage of many years, all seasons, and all sub-divisions across trophic groups. This observation is unsurprising, given the enormous sampling efforts that would be required. It should be mentioned that these studies were usually also tailored to the specific research questions, e.g., regarding specific areas, time-points, species, or trophic groups. However, a broader, systematic coverage of trophic groups, spatial gradients, and temporal changes would be needed to improve ecosystem understanding (Fry 2006; Jennings et al. 2008), particularly so in the spatio-temporally dynamic Baltic Sea (Koho et al. 2021).

*Shortcoming 2: Disconnect of scientific fields and communities* We argue that many of the patterns in the timeline, spatial distribution, and trophic group focus of Baltic SIA studies can be explained in the light of disconnects. As an example, SIA study “hotspots” demonstrate what is possible when regular sampling (e.g., through the integration into monitoring programs) and local expertise in SI ecology (e.g., due to the proximity of research institutes with SIA facilities and expertise) align. Himmerfjärden Bay in ICES SD 27 is a case in point, with an over-proportionally large number of 10% of Baltic SIA studies most likely explained by the close connection of long-standing sampling programs and strong local SIA expertise dating back to the beginning of Baltic SIA work (e.g., Rolff et al. 1993). In contrast, SIA studies are scarce in some other areas, where challenges and research questions for which SIA is in principle ideally suited are immanent but a dedicated local focus on SI ecology may be lacking. Examples include the scarcity of SIA studies addressing changes in terrestrial organic matter inputs (“browning”) (Andersson et al. 2015) in the northern- or of NIS studies in the western Baltic Sea.

A second disconnect concerns “users” of existing SIA methodology versus “developers” at the technological forefront of the field. The Baltic Sea SIA field has included foundation work on the bioaccumulation of contaminants (Broman et al. 1992; Rolff et al. 1993), the development of simultaneous low biomass CNS analysis (Hansen et al. 2009), and the development of correction factors for isotope data derived from preserved macrozoobenthos samples (Umbricht et al. 2018) and lipid content (Kiljunen et al. 2006). Nevertheless, the lag between global and Baltic BSIA and CSIA publication timelines as well as the limited number of highly cited SIA method and foundation papers points to a partial disconnect between the macro-regional and global SIA community.

Thirdly, the low proportion of studies addressing both lower and higher trophic levels, coastal and offshore food webs, or benthic and pelagic systems simultaneously reveal a disconnect between scientific communities, e.g., plankton ecologists and researchers focusing on higher trophic levels or research groups conducting shore-based versus cruise-based expeditions. A narrower focus can be useful to answer questions about specific ecosystem components, but hampers integrated understanding of the shallow Baltic Sea, where benthic–pelagic and coastal offshore coupling play a large role (Griffiths et al. 2017) and bottom-up versus top-down processes are a research priority (Koho et al. 2021).

*Shortcoming 3: Understudied trophic groups* Several groups considered important for Baltic Sea food web functioning presently remain understudied by SIA. Examples include (1) jellyfish, addressed by only five SIA studies to date despite of their putative importance in Baltic Sea food webs (Stoltenberg et al. 2021) contrasting with a rapid increase in studies globally (Choy et al. 2017; Purcell 2018; Chi et al. 2021), (2) marine mammals and seabirds addressed proportionally less than fishes, despite their role as top-level consumers and species of high conservation concern (Sinisalo et al. 2008; Morkūnė et al. 2016), and (3) NIS, addressed by only 11 SIA studies to date, neglecting the trophic ecology of most NIS in the Baltic (Ojaveer et al. 2021). The application of SIA holds particular strengths in studies of fragile, highly mobile, protected and/or non-commercial fauna (e.g., Crawford et al. 2008; Pitt et al. 2008), but this potential has not been fully realized to date.

*Shortcoming 4: Technical considerations* The focus on the “traditional” bulk SIs δ^13^C and δ^15^N and the scarcity of CSIA applications indicate that the Baltic Sea SIA community is currently not exploiting the available toolset as systematically as possible. One example is the small number of BSIA studies including δ^34^S (but see Mittermayr et al. 2014; Kahma et al. 2020), despite its demonstrated applicability to address research questions related to benthic–pelagic coupling. Similarly, the even lower number of BSIA studies focusing on δ^2^H and δ^18^O (Deutsch and Berth 2006; Bartels et al. 2018) contrasts with the usefulness of these isotopes to generate isoscapes and of δ^2^H to contribute to trophic studies (Vander Zanden et al. 2016). Regarding CSIA, the late onset and low number of studies in the Baltic contrast with the rapidly growing number of studies globally (McMahon and McCarthy 2016), and are unfortunate given the demonstrated applicability of CSIA to elucidate organic matter sources at the base of food webs (Larsen et al. 2009) that are so essential for Baltic Sea food web understanding. Likely explanations for these patterns include the higher entry barriers compared to “traditional” BSIA, due to the technological and methodological challenges of δ^2^H and δ^18^O BSIA (Vander Zanden et al. 2016) and CSIA (Nielsen et al. 2018), confounded by the substantially higher cost of the latter. As a result, the number of laboratories able to carry out these analyses and of researchers experienced in the more complex (and still evolving) frameworks for data interpretation is currently limited. However, since the same entry barriers apply globally, but other areas like the United States have nevertheless seen an earlier onset and more commonplace application of these methods, additional, Baltic-specific structural barriers appear to be present.

### Perspective toward the more systematic exploitation of Baltic Sea stable isotope efforts

Considering the substantial advances in all of the fundamental and applied research topics assessed in this review that were driven by SIA studies, the value of expanding on the existing foundations and promoting future SIA efforts is evident. Yet, the current shortcomings also entail that the Baltic Sea SI ecology field has the opportunity to improve. We argue that many of the current shortcomings ultimately relate to the limited extent of integration, e.g., of spatial, temporal, taxonomic or trophic level data, or among scientific communities. On a fundamental level, advancing the field may therefore depend on changes in mentality as much as on specific actions: first, fostering the ambition to address overarching questions about Baltic food webs and ecosystems as a research community, thus moving beyond the current focus on more specific individual study questions, and second, the willingness to actively promote integration. Here, we propose three steps toward these goals.

*Integration *via* increased collaboration* Increased collaboration on the regional level by connecting scientists from different fields (e.g., fisheries biologists, conservation biologists, ecologists, zooarchaeologists, biogeochemists) with active SI ecology groups and laboratories in the Baltic region, and on the global level by an increased integration in international efforts, would create benefits for all parties involved. Dedicated regional multi-partner projects in the tradition of the BONUS program (Snoeijs-Leijonmalm et al. 2017), and systematic contributions to global efforts like the Isobank Consortium (Pauli et al. 2015) and isoscape initiatives (www.waterisotopes.org and its extension isomap; Bowen 2010) would be a way forward. Collaboration with methodologically advanced SIA laboratories both within and outside the Baltic would be a key step to help build capacity and overcome the current technical shortcomings, including the scarcity of CSIA and δ^2^H and δ^18^O BSIA studies related to higher entry barriers relative to traditional BSIA.

*Integration by re-thinking sampling efforts* Integration can also occur at the sampling level by focusing on coordinated efforts beyond individual research groups to provide datasets for more ambitious collaborative studies. This could be accomplished through improved collaboration (see previous section) but also through the integration in Baltic Sea monitoring programs, as previously suggested by Mack et al. (2020). The latter should include dedicated new sampling, but also the more systematic use of the “gold mine” of existing, currently underused sample archives, which are particularly powerful to reconstruct time-series (see e.g., Liénart et al. 2021). Sample sets covering different trophic levels and systems (e.g., benthic versus pelagic, coastal versus offshore) and understudied groups of organisms (e.g., jellyfish, NIS, mammals, birds) with enhanced spatial and temporal resolution would provide the foundation to monitor and understand changes in food web structure and functioning, a research priority in the rapidly changing Baltic Sea (Koho et al. 2021).

*Integration *via* open-access stable isotope databases *In our view, there is another, particularly powerful opportunity for integration at the level of existing but often disconnected data, which is demonstrated by studies combining new with existing datasets to address questions that could otherwise not be tackled (e.g., Savage and Elmgren 2004; Eriksson et al. 2008). What would happen if all primary data from existing studies were compatible and integrated into a single database? The resulting overarching data set would have high-resolution coverage in space, time (including historical and recent periods, seasons), and taxa, and combine all trophic levels and different types of ecosystems. Even without expanding the scope and complexity of individual studies, a host of existing open questions could then be addressed.

On a global scale, similar considerations have led to the US-led initiative “Isobank” (www.isobank.org), aiming to collect and integrate primary SI data from diverse disciplines including organismal biology, ecology, archaeology, and environmental sciences in a single database (Pauli et al. 2015, 2017). Other scientific fields, such as molecular ecology, have already been revolutionized by the implementation of rigorous primary data publication standards and online databases (Imker 2018). We propose that the routine submission of Baltic SI ecology datasets to open-access stable isotope databases holds enormous potential to help overcome current shortcomings and strengthen the Baltic SIA and food web research fields. In particular, with increasing numbers of submitted datasets, this would serve as a bridge for the effective data exchange between researchers, foster the reuse and integration of existing datasets, e.g., in temporal or spatial comparisons, and promote a wider understanding of ecosystem change in the Baltic Sea over time and space.

Since the global “Isobank” is already operational (Pauli et al. 2017), we suggest to start with the systematic submission of new Baltic Sea primary SI datasets and the step-wise retroactive submission of existing datasets from published studies to this database. This should be coupled with the publication of the same datasets in open-access repositories like *Dryad* or *Pangaea*, to further address the low accessibility of Baltic SI datasets. The progress of database and repository contributions (e.g., the proportion of new studies and retroactive submissions) can be monitored against the meta-data collection of all published Baltic SI studies assembled in this systematic review (Table S1, Eglite et al. 2022).

Finally, as long-term perspective, a regionalized database focused on the Baltic Sea, with the establishment of a Baltic Sea plugin in “Isobank” or the implementation of a dedicated “Baltic Isobank”, would align well with other regional efforts, including future science-based monitoring and assessments of the environmental status of food webs under the MSFD D4 and HELCOM HOLAS efforts (HELCOM 2018). Regional efforts such as the dIANA database for paleo-dietary SI data (Etu-Sihvola et al. 2019), the Brasilian SIA-BRA database of δ^13^C and δ^15^N values for terrestrial and aquatic animals (Diniz-Reis et al. 2022), and established Baltic Sea regional databases for biological, oceanographic, and fish stock data in ICES (https://www.ices.dk/data/Pages/default.aspx) and HELCOM (https://helcom.fi/baltic-sea-trends/data-maps/) can serve as models to assess drawbacks and benefits of regionalized efforts.

## Conclusions

The first systematic review of SIA applications in ecological studies in the Baltic Sea identified an active and growing research field that has advanced a range of fundamental and applied research topics, but also revealed structural shortcomings hampering ecosystem-level understanding in the spatio-temporally dynamic Baltic Sea. We argue that a stronger focus on collaboration and integration, including the systematic submission of Baltic Sea primary SI datasets to the global “Isobank” database (Pauli et al. 2017) and long-term perspective of a dedicated macro-regional “Baltic Isobank,” would help to address many of the existing shortcomings. This effort would require large cross-national, multi-partner commitment and investments, but holds the potential for an even larger payoff, in strengthening the Baltic Sea SIA and food web research field, thus benefiting science-based resource management, environmental assessments, and conservation efforts. The effort undertaken here demonstrates the value of macro-regional synthesis, in enhancing access to existing data and supporting the strategic planning of research agendas.

## Supplementary Information

Below is the link to the electronic supplementary material.

Supplementary file1 (PDF 3110 KB)
