# Supplementary material for: Stable isotope analysis in food web research: Systematic review and a vision for the future for the Baltic Sea macro-region
Source: Ambio. 2022 Oct 21;52(2):319–38. doi: 10.1007/s13280-022-01785-1 (PMC9589642; doi:10.1007/s13280-022-01785-1)

Ambio
Supplementary Information
This supplementary information has not been peer reviewed.

Title: **Stable isotope analysis in food web research: systematic review and a vision for the future for the Baltic Sea macro-region**

TABLES

Table S1 3
Table S2 5
Table S3 9

Table S3. Phytoplankton 10
Table S3. Marine macrophytes 11
Table S3. Zooplankton 12
Table S3. Benthic invertebrates 13
Table S3. Jellyfish 14
Table S3. Fish 15
Table S3. Marine mammals 16
Table S3. Waterbirds 17

Table S4 18

FIGURES

Figure S1 19
Figure S2 20
Figure S3 21

Table S1. List of all 12 categories and 43 parameters under which meta-data of SIA studies were extracted, including classification rules for all parameters for which classifications were not self-explanatory. The categories and parameters correspond to the data extraction structure shown in Figure 1.

Category	Parameter(s)	Explanation
Paper	Author, Year, Title, Journal name, Peer-reviewed	All 5 parameters self-explanatory, providing bibliographic information of each paper
Study design	Field/ Experimental/ Both	Classification of studies based on whether they included field work without experimental manipulations, versus experimental work (either laboratory or field), or combined both components
Fundamental knowledge<br><br>(Five topics within this category)	See section "Materials and Methods" for details	
Baltic Sea challenges<br><br>(Four topics within this category)	See section "Materials and Methods" for details	
Trophic groups<br><br>(10 trophic groups in this category)	Classification of studies based on the trophic groups that are covered; all trophic groups represented by species are ticked (i.e., two or more trophic groups can be covered by a study)	
	Seston	Includes organic matter forms and bacterial fraction
	Phytoplankton	Includes all phytoplankton groups (e.g., diatoms, flagellates, cyanobacteria), as well as microzooplankton
	Phytoplankton (Cyanobacteria)	Ticked only if cyanobacteria are covered in the respective studies
	Marine macrophytes	Includes macroalgae and seagrass
	Zooplankton	Includes pelagic crustaceans (e.g., copepods, cladocerans), but not jellyfish (separate entry) and mysids (included under "Benthic invertebrates")
	Benthic invertebrates	For example, benthic crustaceans, molluscs, polychaeta, and also mysids
	Jellyfish	Includes all jellyfish species
	Fish	Includes all fish species (including freshwater and anadromous species, e.g., *Perca fluviatilis*)
	Marine mammals	Includes all marine mammal species
	Waterbirds	Includes seabirds and shorebirds (e.g, *Anas platyrhynchos* duck feeding in coastal systems)
Temporal focus	Year of sampling	Year(s) in which sample(s) for a study was/were taken. Continuous sampling years are displayed as time periods, e.g., the period 2010-2013 reflects sampling in the consecutive years 2010, 2011, 2012, and 2013.
	Month of sampling	Month(s) in which sample(s) for a study was/were taken.
	n of years	Number of years covered by a study (Options: "1", "2", 2+"). Relates directly to the column "Year of sampling"
	Seasonal coverage	Season(s) in which sample(s) for a study was/were taken. Options are winter, spring, summer or autumn
	n of seasons	Number of seasons covered by a study (i.e., numbers between 1 and 4)

Spatial focus	Sampling location (ICES SD)	ICES subdivision(s) (SD) in which sampling for a study took place. SD numbers and names are shown in Figure 3
	Local/ Regional	Spatial focus of studies. “Local” here is defined as a study including samples from one sub-division, “Regional” as a study including two or more sub-divisions. Relates directly to the column “Sampling location”
	Coastal/ Offshore/ Both	Habitat(s) (coastal, offshore or both) covered by studies, based on the description given by the authors in the individual article
	Benthic/ Pelagic/ Benthic-Pelagic	Habitat(s) (benthic, pelagic, or both) covered by studies, based on the description given by the authors in the individual article
	n of trophic groups	Number of trophic groups represented by species included in a study (“1”, “2”, “3” or “3+”). Directly relates to the number of trophic groups that is ticked, without counting seston (which is rather a baseline value than a trophic group). The number here therefore does not reflect an estimated number of trophic levels in the food web.
Stable isotope analysis	Stable isotopes	Stable isotopes included in a study (options for bulk SIA: C, N, S, O, H, labelled C, labelled N; options for compound-specific SIA: C-AAs, N-AAs, C-FAs, labelled C-AAs, labelled N-AAs, labelled C-FAs). “AAs”: amino acids; “FAs”: fatty acids
	SIA application	Options of bulk stable isotope analysis “bulk”, compound-specific stable isotope analysis “compound-specific)”, or “both” for studies including bulk and compound-specific data
	SIA: Labelling/Natural/Both	Options of labelled stable isotopes “Labelling”, natural stable isotopes “Natural”, or “Both” for studies including labelled and natural stable isotope values
Taxonomic focus	Species	Includes all species reported in the main body of the respective publication. Species from supplementary files are not listed systematically here; Species occurring at low samples sizes (e.g. sponges, tunicates) or of terrestrial nature are not covered comprehensively (e.g., leeches, insects)
Study objectives	Objectives	Overarching objective(s) for each study is/are summarized based on title, abstract and introduction.
Online repository	Yes/No	Primary data of study available in an open-access online repository (e.g., Dryad, Pangaea)
	Repository DOI	DOI of a primary dataset, if available
Entry	Original/Update	“Original” refers to inclusion in the table at the time of publication (corresponding to the studies identified in the review. “Updated” is intended for studies added during later updates in an open-access Dryad repository (currently envisioned six months intervals)

Table S2. Complete list of all stable isotope ecology studies in the Baltic Sea identified in this review (n = 164), and the fundamental research topics and Baltic Sea challenges covered by each study (Fundamental knowledge, A, with: A.1 – Food web baseline, A.2 – Food web trophic structure, A.3 – Feeding and Foraging, A.4. – Migration, A.5 – Zooarchaeology; Baltic Sea challenges, B, with: B.1 – Eutrophication, B.2 – Contaminants, B3 – Cyanobacterial blooms, B.4 – Non-indigenous species. The information in this table underlies Figure 3 and Figure S1.

Author	Journal name	A topics	B Topics
Aarnio et al. (2015)	Aquatic Invasions	A.2; A.3	B.4
Aberle & Malzahn (2007)	Oecologia	A.1; A.3; Tech.	Tech.
Adam et al. (2016)	The ISME Journal	A.1	B.3
Ahlbeck Bergendahl et al. (2017)	Journal of Fish Biology	A.1; A.3; A.4	
Almqvist et al. (2010)	Environmental Biology of Fishes	A.2; A.3	B.4
Angerbjörn et al. (2006)	Marine Biology Research	A.3; A.4	
Antanaitis-Jacobs et al. (2009)	Archaeologia Baltica	A.5	
Arroyo et al. (2007)	Journal of Experimental Marine Biology and Ecology	A.3	
Asmala et al. (2019)	Estuaries and Coasts	A.1	B.1
Barrett et al. (2008)	Journal of Archaeological Science	A.5	
Barrett et al. (2011)	Journal of Archaeological Science	A.5	
Bartels et al. (2018)	Ecosystems	A.1	B.1
Bennike et al. (2007)	BOREAS	A.5	
Berezina et al. (2017)	Journal of the Marine Biological Association of the United Kir	A.2; A.3	B.4
Berglund et al. (2001)	Science of the Total Environment	A.2; A.3	B.2
Berglund et al. (2007)	Limnology and Oceanography	A.1; A.2	
Bianchi et al. (2000)	Limnology and Oceanography	A.5	B.1; B.3
Bødker Enghoff (2016)	Environmental Archaeology	A.5	
Boethius & Ahlström (2018)	Journal of Archaeological Science	A.5	
Borgendahl & Westman (2007)	Journal of Paleolimnology	A.5	B.1; B.3
Broman et al. (1992)	Environmental Toxicology and Chemistry	A.2	B.2
Brulinska et al. (2016)	Boreal Environment Research	A.3	
Burreau et al. (2004)	Chemosphere	A.2; A.3	B.2
Burreau et al. (2006)	Science of the Total Environment	A.2	B.2
Byrén et al. (2006)	Marine Ecology Progress Series	A.1	
Corman et al. (2018)	Environmental Monitoring and Assessment	A.2	B.1
Craig et al. (2006)	Journal of Archaeological Science	A.5	
Das et al. (2004)	Marine Ecology Progress Series	A.3	B.2
Deutsch & Berth (2006)	Journal of Applied Ichthyology	A.3	
Deutsch & Voss (2016)	Science of the Total Environment	A.1	B.1
Eglite et al. (2018)	Ecosphere	A.1; A.2; A.3	B.3
Eglite et al. (2019)	Ecology and Evolution	A.3; A.4	B.3
Ek et al. (2018)	Environmental Science & Technology	A.2	B.2
Ek et al. (2019)	PLoS ONE	A.2; Tech.	B.2; Tech.
Ek et al. (2021)	Science of the Total Environment	A.3	B.1; B.2
Eriksson & Lidén (2002)	Journal of Nordic Archaeological Science	A.5	
Eriksson et al. (2004)	Journal of Anthropological Archaeology	A.5	
Eriksson et al. (2008)	Journal of Anthropological Archaeology	A.5	

Table S2 (continued)

Author	Journal name	A topics	B Topics
Fornander et al. (2008)	Journal of Anthropological Archaeology	A.5	
Gagnon et al. (2013)	PLoS ONE	A.1; A.3	B.1
Gagnon et al. (2021)	Limnology and Oceanography	A.2	B.1
Glaubitz et al. (2009)	Environmental Microbiology	A.1	
Glykou et al. (2021)	Quaternary Science Reviews	A.5	
Golubkov et al. (2017)	Journal of Marine Systems	A.1	B.1; B.3
Golubkov et al. (2018)	Marine Pollution Bulletin	A.1	B.1
Golubkov et al. (2019)	Marine Pollution Bulletin	A.1	B.1
Golubkov et al. (2021)	NeoBiota	A.2	B.4
Gorokhova (2018)	Methods in Ecology and Evolution	A.3; Tech.	Tech.
Gorokhova & Hansson (1999)	Canadian Journal of Fisheries and Aquatic Sciences	A.3; Tech.	Tech.
Gorokhova et al. (2005)	Oecologia	A.2; A.3	B.4
Gorokhova et al. (2007)	Limnology and Oceanography	A.3	
Grupe et al. (2009)	Journal of Archaeological Science	A.5	
Hansen et al. (2009)	Rapid Communications in Mass Spectrometry	A.1; Tech.	Tech.
Hansen et al. (2012)	Boreal Environment Research	A.2	
Hanson et al. (2020)	Environmental Toxicology and Pharmacology	A.3	B.2
Hansson et al . (1997)	Ecology	A.1; A.2; A.4	B.1
Hedberg et al. (2021)	Limnology and Oceanography	A.1; Tech.	B.1; Tech.
Herlevi et al. (2018)	Hydrobiologica	A.1; A.2; A.3	B.4
Hobson et al. (2015)	The Auk	A.1; A.3	
Holliland et al. (2012)	Journal of Plankton Research	A.3	B.4
Holmer et al. (2009)	Aquatic Botany	A.1	
Jaatinen et al. (2016)	Frontiers in Zoology	A.3	
Jankowska et al. (2018)	Ecological Indicators	A.1; A.2; A.3	
Jankowska et al. (2019)	Journal of Experimental Marine Biology and Ecology	A.1; A.2; A.3	
Jaschinski et al. (2008a)	Marine Ecology Progress Series	A.1; A.2; A.3	
Jaschinski et al. (2008b)	Limnology and Oceanography	Tech.	Tech.
Jaschinski et al. (2011)	Marine Ecology Progress Series	A.2; A.3	
Javidpour et al. (2016)	Marine Biology	A.3	
Jędruch et al. (2019)	Environmental Monitoring and Assessment	A.1; A.2; A.3	B.2
Jephson et al. (2008)	Marine Environmental Research	A.2; A.3	B.1
Kahma et al. (2020)	Marine Environmental Research	A.1	
Kahma et al. (2021)	Marine Environmental Research	A.2	
Karlson & Mozūraitis (2011)	Harmful Algae	A.1; A.3	B.3
Karlson et al. (2007)	ICES Journal of Marine Science	A.3	B.4
Karlson et al. (2010)	Ecology	A.3	
Karlson et al. (2011)	Oecologia	A.1; A.3	B.4
Karlson et al. (2014)	PLoS ONE	A.1; A.3	B.1; B.3
Karlson et al. (2015)	Scientific Reports	A.3	B.4
Karlson et al. (2018)	Royal Society Open Science	A.3; Tech.	B.2; Tech.
Kiljunen et al. (2006a)	Journal of Applied Ecology	Tech.	Tech.
Kiljunen et al. (2006b)	SIL Proceedings	A.3	
Kiljunen et al. (2008)	Canadian Journal of Fisheries and Aquatic Sciences	A.3	B.2
Kiljunen et al. (2020)	Limnology and Oceanography	A.1; A.2; A.3	
Kolb et al. (2010)	Marine Ecology Progress Series	A.1	B.1

Table S2 (continued)

Author	Journal name	A topics	B Topics
Larsen et al. (2020)	Ecology and Evolution	..1; A.2; A.3; A.4; Tech	Tech.
Laursen et al. (2019)	Ecosystems	A.1	B.1
Ledesma et al. (2020)	Ecology and Evolution	A.3	
Lehtiniemi et al. (2009)	Marine Biology	A.3	
Lesutienė et al. (2007)	Estuarine, Coastal and Shelf Science	A.3	
Lesutiené et al. (2008)	Estuarine, Coastal and Shelf Science	A.1; A.3; A.4	
Lesutienė et al. (2014)	Estuarine, Coastal and Shelf Science	A.1; A.2	B.1; B.3
Lesutienė et al. (2018)	Boreal Environment Research	A.4	B.3
Liénart et al. (2021)	Limnology and Oceanography	A.1; A.2; A.3	B.1; B.3
Limén & Ólafsson (2002)	Marine Biology	A.3	B.3
Linderholm et al. (2014)	Internet Archaeology	A.5	
Ljungberg et al. (2020)	ICES Journal of Marine Science	A.3; Tech.	Tech.
Loick-Wilde et al. (2012)	Limnology and Oceanography	A.1; A.3	B.3
Loick-Wilde et al. (2018)	Limnology and Oceanography	A.1	B.3
Loick-Wilde et al. (2019)	Global change biology	A.1; A.2; A.3	B.3
Magni et al. (2012)	Marine Biology	A.1; A.2	
Marcelina et al. (2018)	Journal of Sea Research	A.1; A.2; A.3	
Meyer-Harms et al. (1999)	Marine Ecology Progress Series	A.1; A.3	B.3
Mittermayr et al. (2014a)	Marine Ecology Progress Series	A.1; A.2; A.3	
Mittermayr et al. (2014b)	Marine Ecology Progress Series	A.1; A.2; A.3	
Morkūnė (2011)	Ekologija	A.2; A.3	
Morkūnė etl al. (2016)	Estuarine, Coastal and Shelf Science	A.1; A.3	
Morkūnė etl al. (2018)	PeerJ	A.1; A.3	
Motwani & Gorokhova (2013)	PLoS ONE	A.1; A.3	B.3
Motwani et al. (2017)	Limnology and Oceanography	A.1; A.3	B.3
Mustamäki et al. (2014)	Environmental Biology of Fishes	A.3	
Nadjafzadeh et al. (2015)	Ibis	A.3	
Nascimento et al. (2011)	Oecologia	A.1; A.3	B.3
Nehlich et al. (2013)	Rapid Communications in Mass Spectrometry	A.1; A.5	
Nfon et al. (2008)	Science of the Total Environment	A.2	B.2
Nfon et al. (2009)	Science of the Total Environment	A.2	B.2
Nielsen et al. (2015)	Marine Ecology Progress Series	A.1; A.3	
Nordström et al. (2009)	Marine Ecology Progress Series	A.2; A.3	
Nordström et al. (2011)	Journal of Experimental Marine Biology and Ecology	A.2; A.3	
Nordström et al. (2016)	Marine Biology Research	A.3	
Ogonowski et al. (2013a)	PLoS ONE	A.3; A.4	
Ogonowski et al. (2013b)	Boreal Environment Research	A.3; A.4	
Ólafsson et al. (2013)	Marine Biology	A.1; A.2; A.3	
Orell et al. (2018)	ICES Journal of Marine Science	A.4	
Orton et al. (2011)	PLoS ONE	A.5	
Persson et al. (2007a)	Environmental Pollution	A.4	B.2
Persson et al. (2007b)	Journal of Fish Biology	A.3	B.2
Piličiauskas et al. (2017)	Archaeological and Anthropological Sciences	A.5	

Table S2 (continued)

Author	Journal name	A topics	B Topics
Rakauskas et al. (2013)	Acta Ichthyologica et Piscatoria	A.2; A.3	B.4
Rakauskas et al. (2020)	Journal of Great Lakes Research	A.2; A.3	B.4
Robson et al. (2012)	Journal of Archaeological Science	A.5	
Rodil et al. (2020)	Frontiers in Marine Science	A.1	
Rolff (2000)	Marine Ecology Progress Series	A.1; A.2	
Rolff & Elmgren (2000)	Marine Ecology Progress Series	A.1; A.2	B.1
Rolff et al. (1993)	Chemosphere	A.2; Tech.	B.2; Tech.
Routti et al. (2005)	Marine Environmental Research	A.3	B.2
Savage (2005)	Ambio	A.1	B.1
Savage & Elmgren (2004)	Ecological Applications	A.1	B.1
Schade et al. (2019)	Marine Ecology Progress Series	A.4; Tech.	Tech.
Scharnweber et al. (2021)	Ecosphere	A.3	
Schubert et al. (2013)	Marine Pollution Bulletin	A.1	B.1
Silberberger et al. (2021)	Frontiers in Marine Science	A.1; A.2; Tech.	Tech.
Sinisalo et al. (2006)	Canadian Journal of Zoology	A.3	
Sinisalo et al. (2008)	Marine Mammal Science	A.3	
Sokołowski et al. (2012)	Estuarine, Coastal and Shelf Science	A.2	
Sommer & Sommer (2004)	Journal of Plankton Research	A.1; A.3	
Sommer et al. (2006)	Journal of Plankton Research	A.1; A.3	B.3
Struck et al. (1998)	Naturwissenschaften	A.1; A.5	B.1; B.3
Suhareva et al. (2021)	Environmental Sciences Europe	A.2; A.3	B.2
Szumiło-Pilarska et al. (2016)	Journal of Trace Elements in Medicine and Biology	A.3	B.2
Szumiło-Pilarska et al. (2017)	Water, Air, & Soil Pollution	A.3	B.2
Szymczak-Żyła et al. (2019)	Paleoceanography and Paleoclimatology	A.5	B.1; B.3
Thormar et al. (2016)	PLoS ONE	A.2	B.1
Torniainen et al. (2014)	ICES Journal of Marine Science	A.3; A.4	
Torniainen et al. (2017a)	Journal of Fish Biology	A.3; A.4	
Torniainen et al. (2017b)	Ecology and Evolution	A.4; Tech.	Tech.
Törnroos et al. (2015)	Journal of Experimental Marine Biology and Ecology	A.1; A.3	
Tverin et al. (2019)	PLoS ONE	A.3	
Ukkonen et al. (2014)	The Holocene	A.5	
Umbricht et al. (2018)	Marine Ecology Progress Series	A.2; Tech.	Tech.
Vuori et al. (2012)	Journal of Fish Biology	A.3; A.4	B.2
Wannicke et al. (2013)	Journal of Marine Systems	A.1	B.3
Webb et al. (2018)	Archaeological and Anthropological Sciences	A.5; Tech.	Tech.
Zakrisson et al. (2014)	Journal of Plankton Research	A.1	B.3
Zguna et al. (2019)	Scientific Reports	A.1; A.3	B.4

Table S3. Complete list of species addressed in the 164 stable isotope ecology studies in the Baltic Sea identified in this review, grouped by the overarching trophic groups phytoplankton, marine macrophytes, pelagic zooplankton, benthic invertebrates, fish, marine mammals and waterbirds, presented as sub-tables on separate pages below. Counts represent the number of studies addressing a specific taxon. The information in this table underlies Figure 4 and 5.

Summary

**Total species count**	239
Benthic invertebrates	75
Fish	52
Phytoplankton	44
Zooplankton	24
Waterbirds	19
Marine macrophytes	16
Marine mammals	6
Jellyfish	3

Table S3. Phytoplankton

	Taxa	Records
Phytoplankton	Nodularia spumigena	17
	Aphanizomenon sp.	15
	Thalassiosira sp.	13
	Dinophysis sp.	8
	Dolichospermum sp.	7
	Chaetoceros sp.	6
	Pseudosolenia calcaravis	4
	Rhodomonas salina	4
	Actinocyclus octonarius	3
	Coscinodiscus sp.	3
	Skeletonema costatum	3
	Aulacoseira islandica	2
	Ceratium sp.	2
	Cyclotella striata	2
	Epithemia turgida	2
	Mesodinium rubrum	2
	Microcystis spp.	2
	Planktothrix sp.	2
	Pseudanabaena sp.	2
	Stephanodiscus neoastraea	2
	Synechococcus sp.	2
	Achnanthes taeniata	1
	Aphanothece sp.	1
	Aphanothece sp.	1
	Chroococcus sp.	1
	Cryptomonas spp.	1
	Dactyliosolen fragillissimus	1
	Dinobryon spp.	1
	Diploneis smithii	1
	Ditylum brightwellii	1
	Dunaliella salina	1
	Gymnodiniales	1
	Heterocapsa triquetra	1
	Nitzschia paleacea	1
	Pauliella taeniata	1
	Pediastrum sp.	1
	Plagioselmis prolonga	1
	Prorocentrum sp.	1
	Prymnesiales sp.	1
	Rhizosolenia hebetata	1
	Scenedesmus sp.	1
	Strombidium spp.	1
	Thalassionema nitzschioides	1
	Tintinnopsis sp.	1

	Taxa	Records
**Total**	**44**	**126**

Table S3. Marine macrophytes

	Taxa	Records
Marine macrophytes	Fucus vesiculosus	13
	Ulva sp.	13
	Zostera marina	13
	Potamogeton pectinatus	12
	Cladophora glomerata	11
	Chara sp.	5
	Myriophyllum spicatum	5
	Polysiphonia sp.	5
	Zannichellia palustris	4
	Ceramium rubrum	3
	Ceratophyllum demersum	2
	Pylaiella littoralis	2
	Ahnfeltia plicata	1
	Delesseria sanguinea	1
	Furcellaria lumbricalis	1
	Phyllophora sp.	1

	Taxa	Records
**Total**	**16**	**92**

Table S3. Zooplankton

	Taxa	Records
Zooplankton	Acartia sp.	25
	Bosmina sp.	18
	Eurytemora affinis	15
	Podon sp.	12
	Evadne nordmanni	11
	Pseudocalanus sp.	10
	Keratella sp.	10
	Temora longicornis	10
	Synchaeta sp.	9
	Centropages sp.	7
	Cercopagis pengoi	5
	Oithona similis	5
	Daphnia sp.	4
	Pleopis polyphemoides	4
	Limnocalanus macrurus	3
	Calanus sp.	2
	Leptodora kindtii	2
	Acanthocyclops viridis	1
	Asplancha sp.	1
	Bryocamptus spp.	1
	Diaphanosoma brachyurum	1
	Mesocyclops leuckarti	1
	Oikopleura dioica	1
	Paracalanus sp.	1

	Taxa	Records
**Total**	**24**	**159**

Table S3. Benthic invertebrates

	Taxa	Records
Benthic invertebrates	Limecola balthica	31
	Gammarus sp.	26
	Mysis sp.	24
	Mytilus sp.	24
	Idotea sp.	19
	Marenzelleria sp.	19
	Monoporeia affinis	19
	Palaemon sp.	14
	Theodoxus fluviatilis	14
	Crangon crangon	14
	Saduria entomon	13
	Cerastoderma sp.	11
	Mya arenaria	11
	Corophium sp.	10
	Hediste diversicolor	10
	Nereis sp.	10
	Pontoporeia femorata	8
	Amphibalanus improvisus	7
	Neomysis integer	7
	Carcinus maenas	6
	Littorina littorea	6
	Mesochra sp.	6
	Radix sp.	6
	Unio sp.	6
	Asterias rubens	5
	Bathyporeia pilosa	5
	Praunus flexuosus	5
	Dreissena polymorpha	5
	Harmothoe sp.	4
	Jaera sp.	4
	Lacuna vincta	4
	Rhithropanopeus harrisii	4
	Rissoa membranacea	4
	Bithynia tentaculata	3
	Capitella capitata	3
	Hydrobia sp.	3
	Lekanesphaera hookeri	3
	Paramysis lacustris	3
	Peringia ulvae	3
	Tachidius discipes	3
	Abra prismatica	2
	Amphithoe rubricata	2
	Cyathura carinata	2
	Erichthonius difformis	2
	Gmelinoides fasciatus	2
	Lymnaea sp.	2
	Microdeutopus gryllotalpa	2
	Potamopyrgus antipodarum	2
	Streblospio shrubsolii	2
	Valvata sp.	2
	Artemia sp.	2
	Candona neglecta	2
	Heterocyprideis sorbyana	2
	Paracyprideis fennica	2
	Adoncholaimus sp.	1
	Akera bullata	1
	Anodonta anatina	1
	Anoplostoma sp.	1
	Arctica islandica	1
	Buccinum undatum	1
	Chelicorophium curvispinum	1
	Conus radiatus	1
	Daptonema sp.	1
	Dexamine spinosa	1
	Diastylis rathkei	1
	Halicryptus spp.	1
	Laophontidae sp.	1
	Lumbriculidae	1
	Magelona sp.	1
	Neptunea antiqua	1
	Nitokra spinipes	1
	Pygospio elegans	1
	Tellina fabula	1
	Theristus sp.	1
	Orconectes limosus	1

	Taxa	Records
Total	75	430

Table S3. Jellyfish

	Taxa	Records
Jellyfish	Aurelia aurita	4
	Cyanea capillata	1
	Haliclystus auricula	1

	Taxa	Records
Total	3	6

Table S3. Fish

	Taxa	Records
Fish	Perca fluviatilis	27
	Clupea harengus	25
	Gadus morhua	20
	Gasterosteus aculeatus	18
	Rutilus rutilus	16
	Salmo salar	15
	Pomatoschistus minutus	12
	Abramis brama	11
	Esox lucius	11
	Syngnathus typhle	11
	Platichthys flesus	11
	Nerophis ophidion	10
	Sprattus sprattus	10
	Ammodytes tobianus	8
	Gymnocephalus cernua	8
	Sander lucioperca	8
	Zoarces viviparus	8
	Pungitius pungitius	7
	Osmerus eperlanus	7
	Belone belone	6
	Pleuronectes platessa	6
	Spinachia spinachia	6
	Neogobius melanostomus	6
	Coregonus lavaretus	5
	Gobius niger	4
	Myoxocephalus quadricornis	4
	Alburnus alburnus	4
	Vimba vimba	4
	Anguilla anguilla	3
	Blicca bjoerkna	3
	Gobiusculus flavescens	3
	Salmo trutta	3
	Leuciscus sp.	3
	Limanda limanda	2
	Lota lota	2
	Scardinius erythrophthalmus	2
	Syngnathus rostellatus	2
	Tinca tinca	2
	Coregonus albula	1
	Cyclopterus lumpus	1
	Hyperoplus lanceolatus	1
	Melanogrammus aeglefinus	1
	Merlangius merlangus	1
	Myoxocephalus scorpius	1
	Myxine glutinosa	1
	Perccottus glenii	1
	Pholis gunnelus	1
	Platichthys solemdali	1
	Pomatoschistus microps	1
	Scophthalmus maximus	1
	Taurulus bubalis	1
	Silurus glanis	1

	Taxa	Records
Total	52	327

Table S3. Marine mammals

	Taxa	Records
Marine mammals	Halichoerus grypus	13
	Pusa hispida	13
	Pagophilus groenlandicus	11
	Phocoena phocoena	7
	Phoca vitulina	4
	Lagenorhynchus albirostris	1

	Taxa	Records
Total	6	49

Table S3. Waterbirds

	Taxa	Records
Waterbirds	Larus sp.	9
	Phalacrocorax carbo	6
	Somateria mollissima	6
	Anas platyrhynchos	5
	Ardea cinerea	2
	Bucephala clangula	2
	Gavia arctica	2
	Haliaeetus albicilla	2
	Melanitta fusca	2
	Mergus merganser	2
	Podiceps cristatus	2
	Tringa nebularia	2
	Uria aalge	2
	Alca torda	1
	Anser sp.	1
	Chroicocephalus ridibundus	1
	Clangula hyemalis	1
	Fulica atra	1
	Gavia stellata	1

	Taxa	Records
Total	19	50

Table S4. List of non-peer-reviewed academic theses (n = 10), conference papers (n = 2), reports (n = 1), and preprints (n = 2) with a focus on stable isotope ecology in the Baltic Sea. The search results are not checked for completeness and do not represent a systematic review.

Paper	Year	Title	Available
Agurto (2007)	2007	Assessing mesozooplankton trophic levels in the Baltic Sea and North Sea: A stable isotope study	Doctoral thesis / Kiel University
Danielsson et al. (2015)	2015	Carbon and nitrogen stable isotopes within the Swedish national monitoring of contaminants in marine biota	Report/ Swedish Museum of Natural History
Hobbie et al. (1990)	1990	Sewage derived 15N in the Baltic traced in fucus	Conference paper/ EOS
Karlson et al. (2021)	2021	Trophic position derived from amino-acid nitrogen isotopes reflect physiological status of both predator and prey over four decades	Preprint/ bioRxiv
Kiljunen et al. (2018)	2018	Movements of Individual Salmon (*Salmo salar*) in the Baltic Sea Revealed by Stable Isotopes	Conference paper / European Conference on Computational Biology
Lehosmaa (2014)	2014	Dietary differences between Baltic ringed seals (*Phoca hispida botnica)* based on stable isotope and fatty acid analyses	Master thesis / University of Jyväskylä
Lesutienė (2009)	2009	Food web of the Curonian Lagoon: organic matter sources and feeding of mysids	Doctoral thesis/ Klaipeda University
Lienart et al. (2021)	2021	Spatio-Temporal Variation in Stable Isotope and Elemental Composition of Key-Species Reflect Environmental changes in the Baltic Sea	Preprint/ Biogeochemistry
Mänttäri (2011)	2011	Hallien (*Halichoerus grypus*) ja itämerennorppien (*Phoca hispida botnica*) ravinnonkäyttö Perämerellä	Master thesis / University of Jyväskylä
Mohm (2014)	2014	Feeding ecology of Baltic cod assessed by stable isotope analysis	Bachelor thesis / Kiel University
Mohm (2016)	2016	Trophic dynamics of Baltic fish species after the 2014 Major Baltic Inflow event	Master thesis / Kiel University
Morkūnė (2017)	2017	Food web of the Lithuanian Baltic Sea coastal zone: structure and organic matter flows	Doctoral thesis/ Klaipeda University
Priester (2018)	2018	Otolith time machine: 40 year history of Baltic cod feeding ecology reconstructed with otolith protein C, N and S stable isotope analysis	Bachelor thesis / Kiel University
Sokołowski (2009)	2009	Tracing the flow of organic matter based upon dual stable isotope technique, and trophic transfer of trace metals in benthic food web of the Gulf of Gdańsk (southern Baltic Sea)	Habilitation thesis/University of Gdańsk Press
Urban (2016)	2016	Unlocking the potential of biological sample archives: benthic-pelagic feeding of Baltic cod assessed by otholith protein amino-acid specific stable isotope analysis	Bachelor thesis / Kiel University

Figure S1. Coverage of A.) fundamental research topics ("Fundamental knowledge") and B.) applied topics ("Baltic Sea challenges") by stable isotope ecology studies in the Baltic Sea. Numbers above the bars reflect the count of studies from the overall study pool (n = 164) addressing each topic. This figure complements manuscript Figure 3 providing regionally resolved information but not overall numbers.

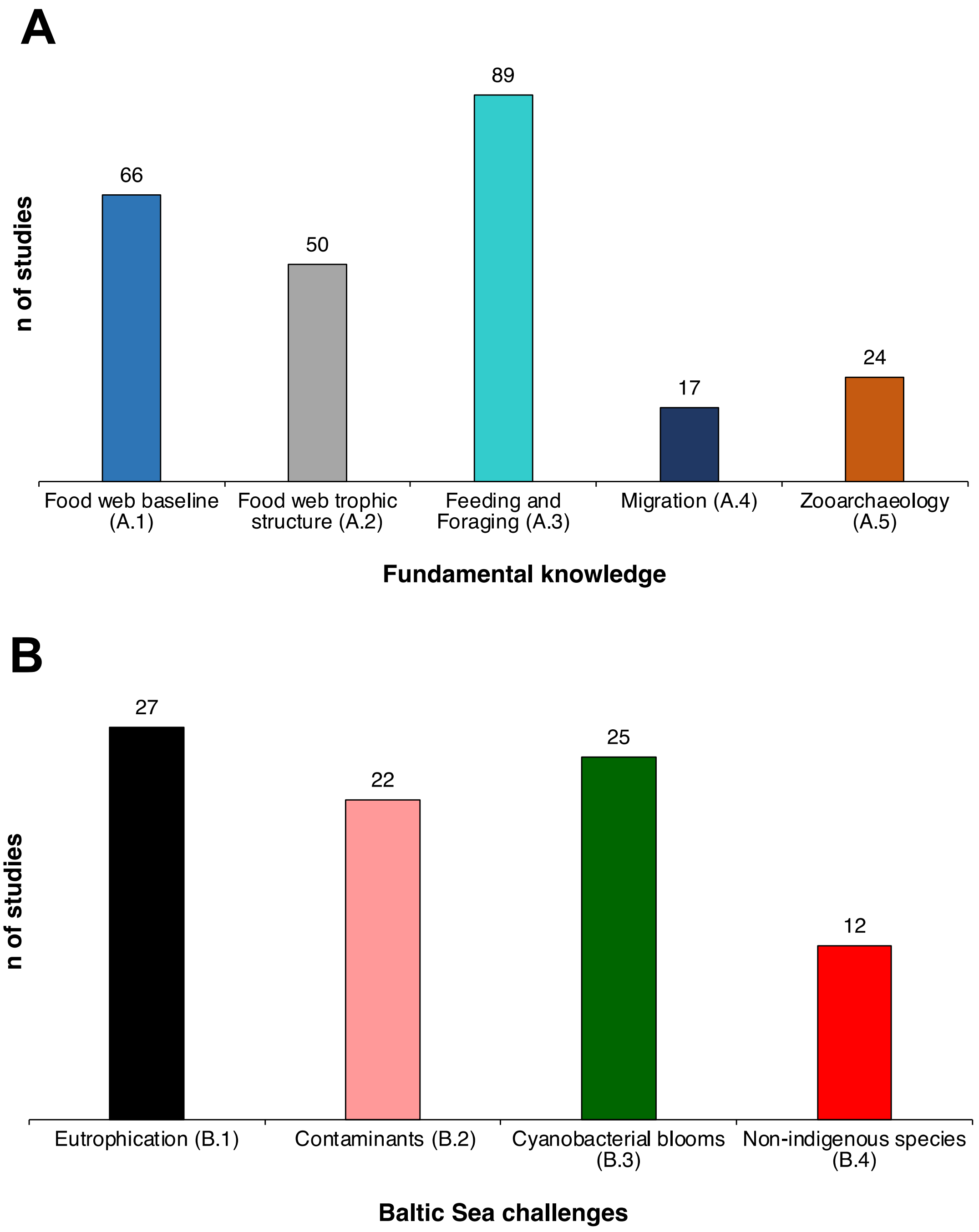

Figure S2. Specific combinations of stable isotopes used in Baltic Sea stable isotope ecology studies. Numbers above the bars represent the count of studies applying specific stable isotopes. Stable isotopes marked with AAs (= amino acids) and FAs (= fatty acids) represent compound specific analyses, all others bulk analyses.

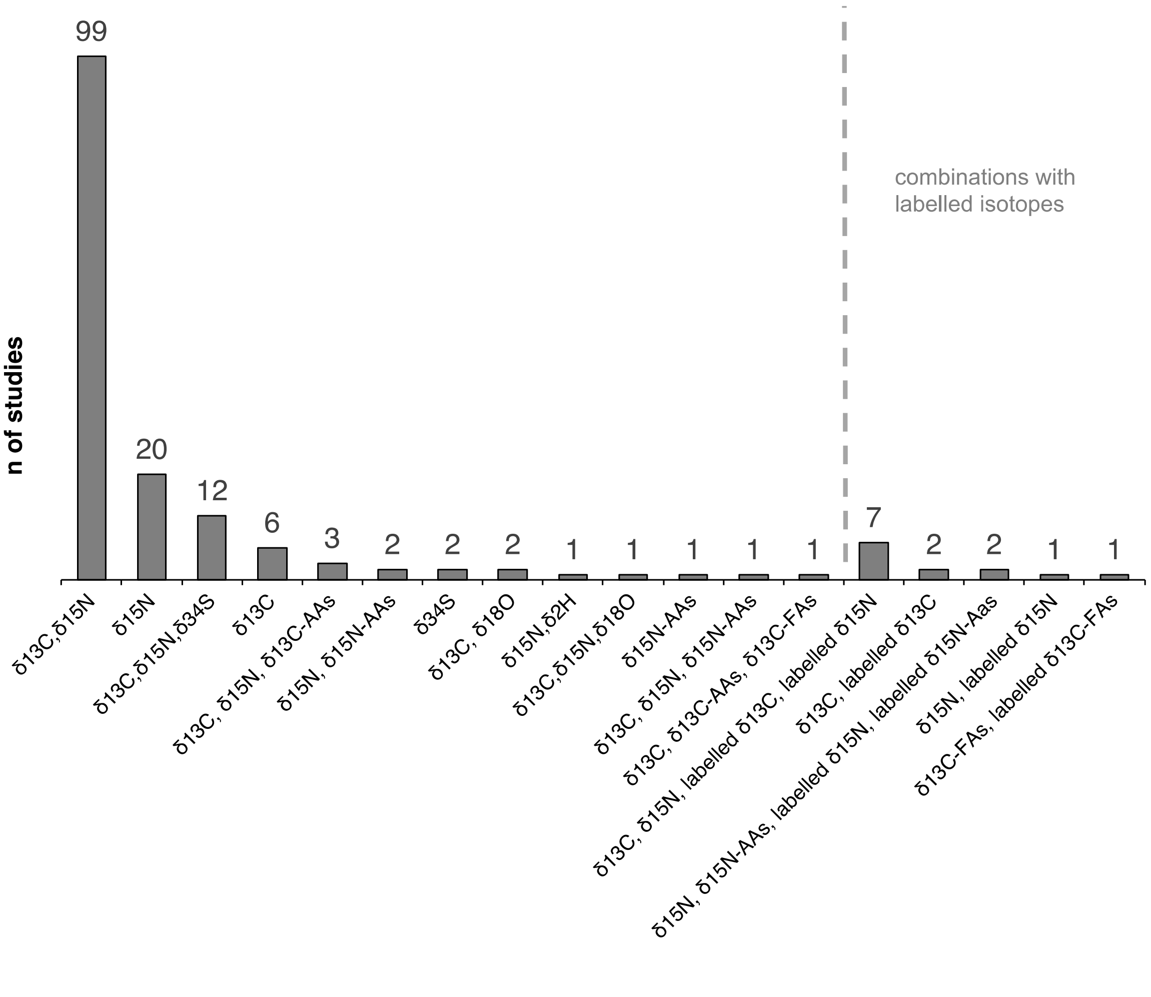

Figure S3. Temporal comparison of the relative importance of different topics covered by stable isotope studies during the time periods 1999–2009 and 2010–2021, for the pool of studies focusing on A) fundamental knowledge topics (n = 240) and B) applied topics (n = 85).

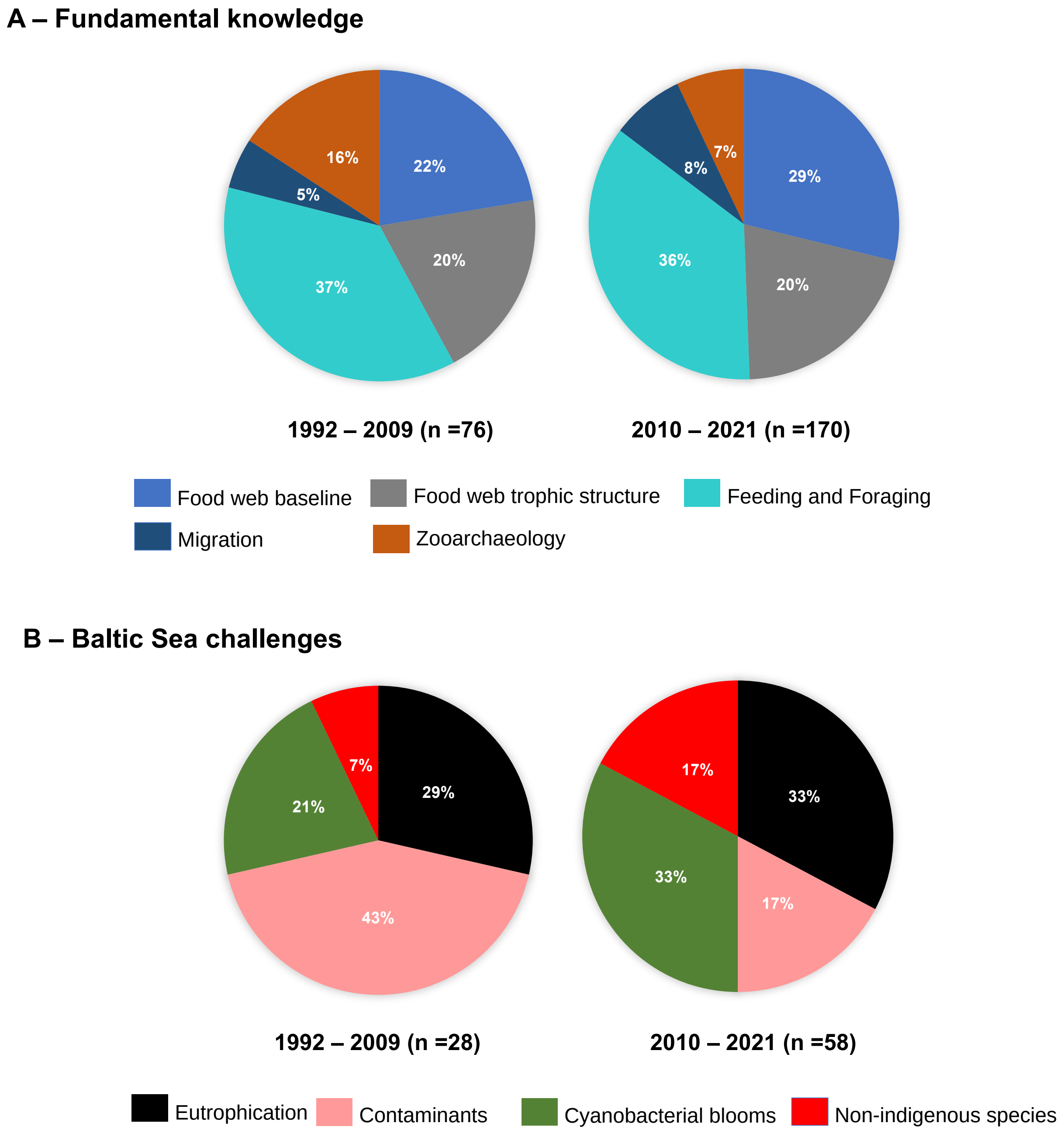

Supplement: Supplementary file 1 — Supplementary file1 (PDF 3110 KB) [file 13280_2022_1785_MOESM1_ESM.pdf]
